# Assessment of Sublethal and Transgenerational Effects of Pirimicarb on the Wheat Aphids *Rhopalosiphum padi* and *Sitobion avenae*

**DOI:** 10.1371/journal.pone.0128936

**Published:** 2015-06-29

**Authors:** Da Xiao, Ting Yang, Nicolas Desneux, Peng Han, Xiwu Gao

**Affiliations:** 1 Department of Entomology, China Agricultural University, Beijing, 100193, China; 2 French National Institute for Agricultural Research (INRA), Univ. Nice Sophia Antipolis, CNRS, UMR 1355-7254 Institut Sophia Agrobiotech, 06903, Sophia-Antipolis, France; Institute of Vegetables and Flowers, Chinese Academy of Agricultural Science, CHINA

## Abstract

The wheat aphids, *Rhopalosiphum padi* (Linnaeus) and *Sitobion avenae* (Fabricius), are key pests on wheat crops worldwide. Management practices rely primarily on insecticides. The pirimicarb (carbamate) is used extensively as an effective insecticide to control these two aphids. In addition to the mortality caused by pirimicarb, various sublethal effects may occur in aphids when exposed to low lethal or sublethal doses. Understanding the general effect of pirimicarb on aphids could help increasing rational use of this insecticide. Under laboratory conditions, we assessed the sublethal effects of a low lethal concentration of pirimicarb (LC_25_) on biological traits and acetylcholinesterase (AChE) activity of *R*. *padi* and *S*. *avenae*. Both direct and transgenerational effects, i.e. on parent and the F1 generations were assessed, respectively. We found that *R*. *padi* and *S*. *avenae* responded differentially to the LC_25_ of pirimicarb. The parent generation of *R*. *padi* showed a 39% decrease in fecundity and multiple transgenerational effects were observed in the F1 generation; overall juvenile development, reproductive period, adult longevity and lifespan were longer than those of the control group. By contrast, LC_25_ of pirimicarb showed almost no effects on *S*. *avenae* biological traits in both the parent and F1 generations; only the pre-reproductive duration was reduced in F1 generations. Demographic parameter estimates (e.g. *r_m_*) showed similar trend, i.e. significant negative effect on *R*. *padi* population growth and no effect on *S*. *avenae*. However, AChE activity decreased in both *R*. *padi* and *S*. *avenae* treated by the LC_25_ of pirimicarb. We demonstrated sublethal and transgenerational effects of pirimicarb in the two wheat aphid species; it hinted at the importance of considering sublethal effects (including hormesis) of pirimicarb for optimizing Integrated Pest Management (IPM) of wheat aphids.

## Introduction

The bird cherry-oat aphid, *Rhopalosiphum padi* (Linnaeus) and grain aphid, *Sitobion avenae* (Fabricius) are destructive pests of wheat, sorghum and other small grain crops worldwide [1–3]. These two species usually coexist at the late stage of wheat growth in China [4], causing direct damage through feeding. Moreover, they also serve as vectors of barley yellow dwarf virus [5]. Outbreaks of these two aphids lead to severe yield losses [6]

In China, insecticides application is primary strategy to control aphids. Organophosphate and carbamate insecticides are extensively used for wheat aphid control [7]. Carbamate insecticides are esters of carbamic acid, and they inhibit hydrolysis of neurotransmitter acetylcholine (ACh) by acetylcholinesterase (AChE) resulting in the disruption of normal nervous system functions. Pirimicarb is a selective carbamate aphicide and mainly used for management of wheat aphid [8, 9]; it has been used for the control of wheat aphids since the 1990s in China [10].

In addition to their lethal effects, insecticides may also impair various key biological traits of aphids in the exposed insects through sublethal effects [11]. Sublethal effects are defined as physiological and/or behavioral effects on individuals that survived from exposure to a pesticide at low or sublethal concentrations/doses [11]. Sublethal effects of insecticides could affect population dynamics through impaired behavior and physiological traits, such as life span, development rates, fertility and fecundity [12–16]. Studies have demonstrated that exposure to lethal or sublethal doses/concentrations of pesticides could reduce insect longevity and fecundity [17–21]. Therefore, assessment of sublethal effects is crucial to acquiring knowledge on global insecticide efficacy on insect pests, as well as on possible selectivity towards non-target organisms [22, 23]. In addition, sublethal insecticide exposure could be assessed using specific biomarkers e.g. AChE and glutathione S-transferase (GST) activity [24–27]. Numerous studies have focused on insect AChE because it is target of organophosphates and carbamates insecticides, which are two major pesticide classes used worldwide for pest management [28].

In China, *R*. *padi* and *S*. *avenae* are susceptible to pirimicarb according to established susceptible base line; thus pirimicarb is used efficiently to control these aphids [29, 30]. However, the extensive use of this insecticide and possible inappropriate and/or sub-optimal applications may promote the development of resistances as reported for many other pest-pesticide couples [31–34]. In addition, abiotic disturbances are known to affect ecological interactions among species [35]; an insecticide-mediated shift in ecological dominance between the wheat aphids might occur through the persistence of low pesticide concentrations in fields. Understanding the general effect of pirimicarb on the aphids would help increase rational use of this insecticide in wheat and also increase its sustainable use. In this context, we investigated the direct effect of pirimicarb on wheat aphids under a low lethal concentration (LC_25_) on exposed individuals i.e. the parent generation, as well as the transgenerational effect on the subsequent generation (F1) [33]. Assessment of such effects could be highly relevant for *R*. *padi* and *S*. *avenae* that have high reproductive rates and short life cycles. In addition, the activity of AChE was characterized to complement the assessment of effects of pirimicarb on wheat aphids. The LC_25_ was used because such low concentration may occur in fields when insecticides degrade following initial application ([e.g. see [36]).

## Materials and Methods

### Insects

Colonies of *R*. *padi* and *S*. *avenae* were initiated from apterous aphid clones collected from a wheat field of the Agricultural Experiment Station, China Agricultural University (N40°03’, E116°28’). Colonies of the two aphid species were maintained in the laboratory without insecticides exposure since May 2005. They were reared on wheat seedlings under laboratory conditions (18–25°C, relative humidity 60 ± 10% and a photoperiod of 17:7 L:D) according to Lu and Gao (2007)[4].

### Insecticides and chemicals

Pirimicarb (95% technical grade) was obtained from Wuxi Ruize Chemical Co. Ltd, China. For assessment of the AChE activity, S-Acetylthiocholine iodide (ATchI) and 5, 5’-Dithiobis (2-nitrobenzoic acid) (DTNB) were purchased from Sigma (St. Louis. MO, USA). Bovine serum albumin (BSA) was purchased from Tongzheng Biological Company (Beijing, China) and was used as standard for assessment of AChE activity.

### Insecticide bioassays

Insecticide toxicity was assayed following the method of exposure to pesticide residues in glass tubes (diameter: 2 cm, length: 5.2 cm) [29]. Pirimicarb was diluted into six different concentrations in analytical grade acetone. An aliquot of 200 μl insecticide-acetone solution was applied to each tube. These tubes were immediately rotated using a micro-rotator (American Wheaton Company) until solutions were dried. Twenty aphids were treated for each concentration with three replications. Controls were treated with acetone only. The aphids were then reared under laboratory conditions and mortality was checked after three hours. After this 3-h period the mortality was assessed; aphids that did not move legs when touched with a fine brush (no reflex movement) were considered dead [37]. The results of testing the six pirimicarb concentrations were used to estimate the LC_25_ for the following experiments on sublethal effects of pirimicarb.

### Impact of LC_25_ pirimicarb on biological traits in parent generation of the aphids

The assays were conducted on wheat seedling stage using life table construction. Wheat seedlings were grown in plastic dishes (diameter: 4 cm) with a slice of wringing paper-filter put at the bottom of plastic dish to keep humidity under laboratory conditions. Pirimicarb was prepared in acetone and diluted to the LC_25_ with distilled water containing 0.05% (v/v) Triton X-100. The adult aphids fed on wheat seedlings that were treated with LC_25_ of pirimicarb for 10 s following the method of leaf dipping [37]. Mortality was calculated at 24 h post treatment and the survivals were gently moved into the new wheat seedlings without any insecticide. Survival aphids were kept in plastic dish individually and each plastic dish was sealed with fitted ventilated plastic tubes in order to prevent aphid escape. The number of progeny nymphs was recorded and then removed daily until the adult aphid was dead. Wheat seedlings were replaced every week during the experiment period. Three replicates were carried out with twenty-five aphids for each replicate. The aphids in control group fed on the seedlings treated with distilled water containing 0.05% (v/v) Triton X-100.

### Transgenerational sublethal effects of pirimicarb in F1 generation of the aphids

Using the same experimental protocol as described above, we exposed adult aphids to LC_25_ pirimicarb. Five survival adult aphids were transferred into the new wheat seedling after treatment with LC_25_ of pirimicarb at 24 h post treatment, and then maintained in the plastic dishes as described above. At 24 h after the transfer, all of the aphids were removed leaving only one neonate nymph in the wheat seedling. Thereafter, we recorded (i) the duration of development, (ii) the numbers of survivors at different development stages, and (iii) the numbers of nymphs produced per aphid every day. These recorded data were used to estimate (i) duration of nymphs or generation (in days), (ii) pre-reproductive or reproductive period (in days), (iii) longevity of adults (in days), and (iv) total number of nymphs laid per aphid during its life (cited as fecundity hereafter). During the reproductive period, the newborn nymphs were counted and then removed daily. Data from alate aphids were not included in the analysis. Three replicates were carried out with twenty-five aphids for each replicate. The aphids in control group fed on the seedlings treated with distilled water containing 0.05% (v/v) Triton X-100.

The life-table parameters, which include the (i) net reproductive rate (*R*_*0*_ = ∑*l*_*x*_ *m*_*x*_): the population growth rate per generation with regard to the number of female offspring produced per female, (ii) mean generation time (*T* = ∑*xl*_*x*_ *m*_*x*_ /*R*_*0*_): the average interval separating births from one generation to the next, (iii) the intrinsic rate of increase (*r*_*m*_ = ln(*R*_*0*_)/*T*): the maximum exponential increase rate in a population growing within defined physical conditions and (iv) population doubling time (*Dt* = ln(2)/ *r*_*m*_): the time required by a population, when growing exponentially, to double when it increase as a given *r*_*m*_ were calculated as follows for control and treatment. The age-specific survival rate (lx) is the proportion of individuals in the initial cohort alive at age x time (days), and the age-specific fecundity (mx) is the mean number of female progeny produced per female alive at the age interval, x days.

### Determination of AChE activity

AChE activity was assessed with ATChI as substrates according to the method of Ellman et al. (1961) [38] and Gao (1987) [39]. The enzyme preparation was replicated three times and each replicate was prepared by homogenizing 60 wingless adult aphids. They were placed in 1ml ice-cold phosphate buffer (0.04 M, pH 7.5, containing 0.1% (v/v) Triton X-100). The homogenates were centrifuged at 4°C, 10, 800 g (Eppendorf centrifuge 5417R, Germany) for 30 min, and the supernatant was used for subsequent AChE activity assay. The assay mixture contain 100 μl of enzyme preparation, 100 μl of substrate solution (5 mM). The reaction was stopped by the addition of 3.6 ml DNTB (0.125 mM) with 40% ethanol after incubating at 30°C for 15 min. The optical density (OD) at 412 nm was measured using spectrophotometer (Lambda Bio 40, PE, USA). The control samples contained no enzyme during the incubation. AChE activity was based on protein content which was determined by the method of Bradford (1976) [40], using BSA as the standard. AChE activities were expressed as nmol ATCh hydrolyzed per min per mg protein using the extinction coefficient of 1.36×10^4^ M^-1^ cm^-1^.

### Data analysis

Datasets were first tested for normality and homogeneity of variance using the Kolmogorov-Smirnov and the Cochran tests respectively and were transformed if necessary. The LC_25_ of pirimicarb for *R*. *padi* and *S*. *avenae* was determined using a log-probit model [41]. The life table and population growth datasets were subjected to t-tests to compare pirimicarb-exposed groups to respective control groups (GraphPad Software, San Diego, CA, USA).

## Results

### LC_25_ of pirimicarb

The LC_25_ of pirimicarb were 0.29 μg mL^-1^ and 0.60 μg mL^-1^ for *R*. *padi* and *S*. *avenae*, respectively (Table 1). When these concentrations were used for subsequent assessment of sublethal effects, the corrected mortality were 25.8% and 23.2% for *R*. *padi* and *S*. *avenae*, respectively.

**Table 1:** LC_25_ values and mortality for pirimicarb toxicity on *R*. *padi* and *S*. *avenae* after 3 h exposure to treated glass tubes.

Insect	Slope ± SE^a^	LC_50_ ^b^	LC_25_ ^b^	χ^2^ ^c^	*P* values	Mortality^d^(%)
*R*. *padi*	1.43 ± 0.27	0.53(0.40–0.64)	0.29 (0.18–0.39)	1.29	0.206	25.80
*S*. *avenae*	1.57 ± 0.38	1.36(0.78–1.93)	0.60 (0.25–9.49)	2.44	0.584	23.22

^a^ SE = standard error.

^b^ Expressed in μg mL^-1^; 95% CI of LC_25_ are given in bracket.

^c^ Chi-square testing linearity of dose-mortality responses.

^d^ LC_25_ induced mortality.

### Longevity and fecundity of parent generation of *R*. *padi* and *S*. *avenae*

The longevity of *R*. *padi* and *S*. *avenae* from parent generation was not affected by the LC_25_ of pirimicarb when compared to their respective control (Table 2, *P* = 0.847 and *P* = 0.972, respectively). The fecundity of *R*. *padi* exposed to pirimicarb was significantly lower than that in the control group (*P* < 0.001) (Table 2). However, no significant difference was found in the fecundity of *S*. *avenae* exposed to pirimicarb (*P* = 0.694) (Table 2).

**Table 2:** Effect of LC_25_ of pirimicarb on longevity and fecundity of *R*. *padi* and *S*. *avenae* from the parent generation.

Insect	Longevity of adults (days ± SD)					Number of nymphs laid per aphid ± SD				
	Control	Pirimicarb	*P*	*t*	df	Control	Pirimicarb	*P*	*t*	df
*R*. *padi*	12.84 ± 1.03	13.29 ± 2.12	0.847	0.194	47	49.44 ± 3.50	**29.79 ± 5.51**	<0.001	4.162	47
*S*. *avenae*	14.41 ± 0.98	13.70 ± 1.88	0.972	0.035	67	14.74 ± 1.61	14.48 ± 1.77	0.694	0.395	67

The values (mean ± SD) in bold text are significantly different when compared to their respective control (t-test) at *P* < 0.05.

### Fecundity of *R*. *padi* and *S*. *avenae* in F1 generation

There was no significant impact of pirimicarb on total fecundity in F1 generation of *R*. *padi* (*P* = 0.757), whereas daily fecundity significantly decreased after exposed to pirimicarb (*P* < 0.001) (Table 3). There was no significant impact of pirimicarb on total fecundity (*P* = 0.690) and daily fecundity (*P* = 0.745) in F1 generation of *S*. *avenae* (Table 3).

**Table 3:** Effect of LC_25_ of pirimicarb on fecundity of *R*. *padi* and *S*. *avenae* from the F1 generation.

Insect	Number of nymphs laid per aphid ± SD					Number of nymphs laid per aphid daily ± SD				
	Control	Pirimicarb	*P*	*t*	df	Control	Pirimicarb	*P*	*t*	df
*R*. *padi*	60.50 ± 3.74	62.38 ± 4.60	0.758	0.314	46	5.26 ± 0.28	**3.63 ± 0.26**	<0.001	4.250	46
*S*. *avenae*	23.44 ± 1.99	22.12 ± 2.73	0.690	0.400	86	1.67 ± 0.09	1.61 ± 0.13	0.745	0.327	86

The values (mean ± SD) in bold text are significantly different when compared to their respective control (t-test) at *P* < 0.05.

### Development of *R*. *padi* and *S*. *avenae* in F1 generation

**Fig 1:**
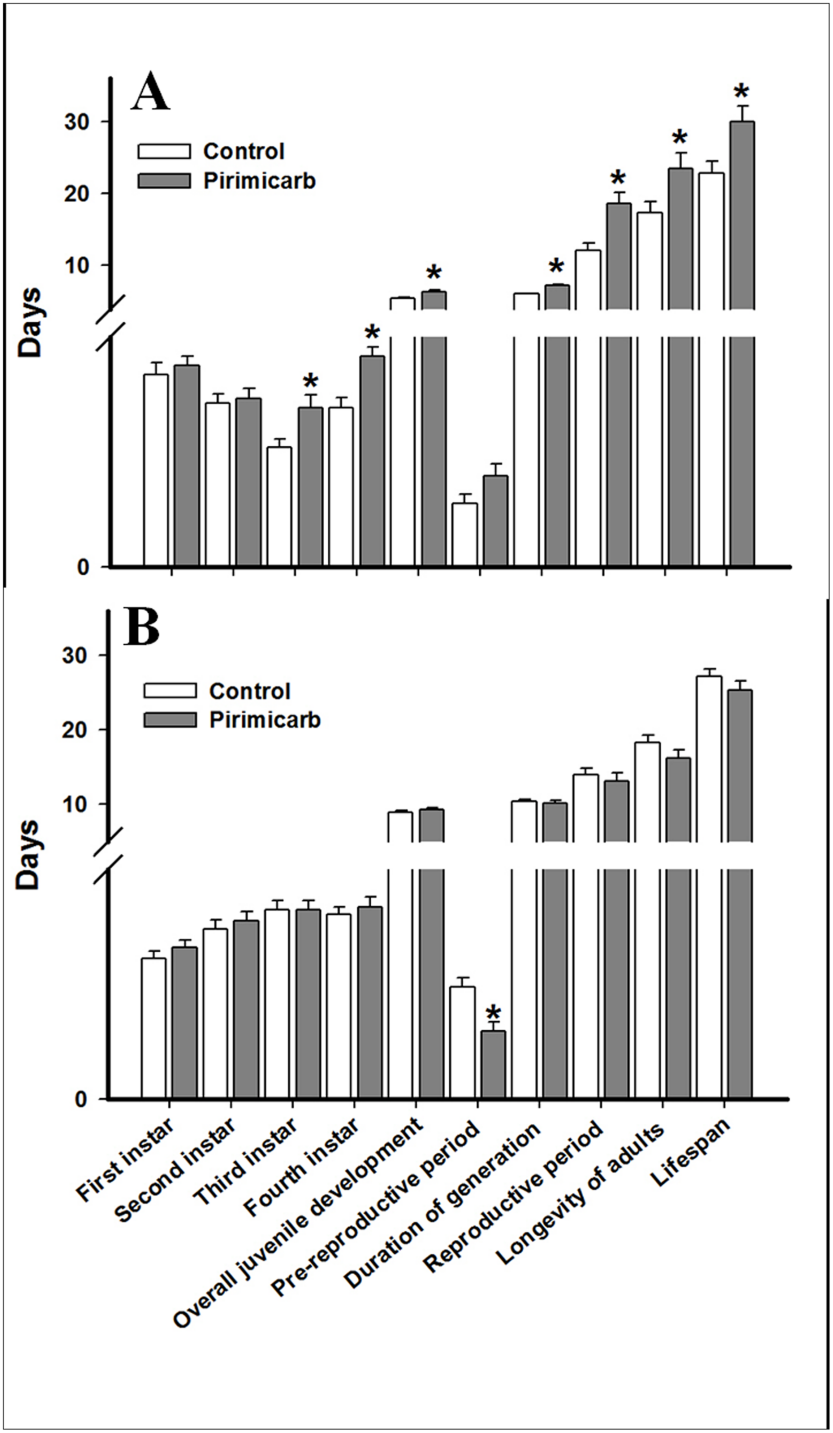
Effects of LC_25_ of pirimicarb on biological traits of *R*. *padi* (A) and *S*. *avenae* (B) in the F1 generation. Asterisk on the bars of the histogram indicate significant differences between treatment and respective control (t-test) at *P* < 0.05.

The effects of pirimicarb exposure on development in F1 generation of *R*. *padi* were reported in Fig 1A. The development time of the first and second instar of *R*. *padi* were not affected, while the development time of the third instar was significantly longer (1.38 ± 0.11 days) after pirimicarb exposure than in the control group (1.04 ± 0.07 days) (t = 2.599, *P* = 0.013). The development time of the fourth instar of *R*. *padi* was significantly longer (1.83 ± 0.08) after exposed to pirimicarb than that of control (1.38 ± 0.09 days) (t = 3.817, *P* < 0.001). The overall juvenile development of *R*. *padi* was significantly longer (6.42 ± 0.18 days) in the treatment than that of the control (5.50 ± 0.14 days) (t = 3.943, *P* < 0.001). In general, the duration of generation of *R*. *padi* significantly increased (7.21 ± 0.23 days) after exposure to pirimicarb compared to the control (6.04 ± 0.11 days) (t = 4.639, *P* < 0.001). Exposure to LC_25_ of pirimicarb significantly increased the reproductive period of *R*. *padi* (t = 3.621, *P* < 0.001). The adult longevity of *R*. *padi* was also significantly longer (23.54 ± 2.10 days) after exposed to pirimicarb than that of the control (17.38 ± 1.54 days) (t = 2.370, *P* = 0.022). The lifespan of *R*. *padi* was significantly longer (29.96 ± 2.16 days) after pirimicarb exposure than that of the control (22.88 ± 1.58 days) (t = 2.644, *P* = 0.011). When *S*. *avenae* was exposed to LC_25_ of pirimicarb, no biological traits were significantly affected compared to their respective control, except for the pre-productive period. The pre-productive period of *S*. *avenae* was significantly shorter (0.88 ± 0.12 days) after exposure to pirimicarb than that of the control (1.46 ± 0.12 days) (t = 3.243, *P* = 0.002) (Fig 1B).

### Demographic parameters of *R*. *padi* and *S*. *avenae* when exposed to LC_25_ pirimicarb

The intrinsic rate of increase (*r*_m_) of *R*. *padi* were significantly lower when exposed to LC_25_ of pirimicarb when compared to the control (Table 4). In addition, the mean generation time (*T*) and population doubling time (*Dt*) of *R*. *padi* were significantly higher than control when exposed to LC_25_ of pirimicarb. By contrast, exposure to LC_25_ of pirimicarb showed no significant effect on *S*. *avenae* demographic parameters (Table 5).

**Table 4:** Effect of LC_25_ of pirimicarb on demographic parameters of *R*. *padi*.

Demographic Parameter	*R*. *padi*		Statistics		
	Control	Treatment	*P*	*t*	df
Net reproductive rate: *R_0_*	56.20 ± 6.34	55.50 ± 5.74	0.077	3.226	46
Intrinsic rate of increase: *r_m_*	0.36 ± 0.01	**0.27 ± 0.02**	< 0.001	6.835	46
Mean generation time: *T* (day)	11.33 ± 0.67	**14.75 ± 0.54**	< 0.001	7.290	46
Population doubling time: *Dt* (day)	1.95 ± 0.11	**2.55 ± 0.21**	< 0.001	6.447	46

The values (mean ± SD) in bold text are significantly different when compared to their respective control (t-test) at *P* < 0.05.

**Table 5:** Effect of LC_25_ of pirimicarb on demographic parameters of *S*. *avenae*.

Demographic Parameter	*S*. *avenae*		Statistics		
	Control	Treatment	*P*	*t*	df
Net reproductive rate: *R_0_*	20.19 ± 2.85	19.10 ± 1.39	0.648	0.504	86
Intrinsic rate of increase: *r_m_*	0.18 ± 0.01	0.18 ± 0.01	0.373	0.924	86
Mean generation time: *T* (day)	16.44 ± 0.74	16.01 ± 0.69	0.831	0.204	86
Population doubling time: *Dt* (day)	3.79 ± 0.13	3.76 ± 0.09	0.596	0.617	86

The values (mean ± SD) in bold text are significantly different when compared to their respective control (t-test) at *P* < 0.05.

### AChE activity of parent generation in *R*. *padi* and *S*. *avenae*

**Fig 2:**
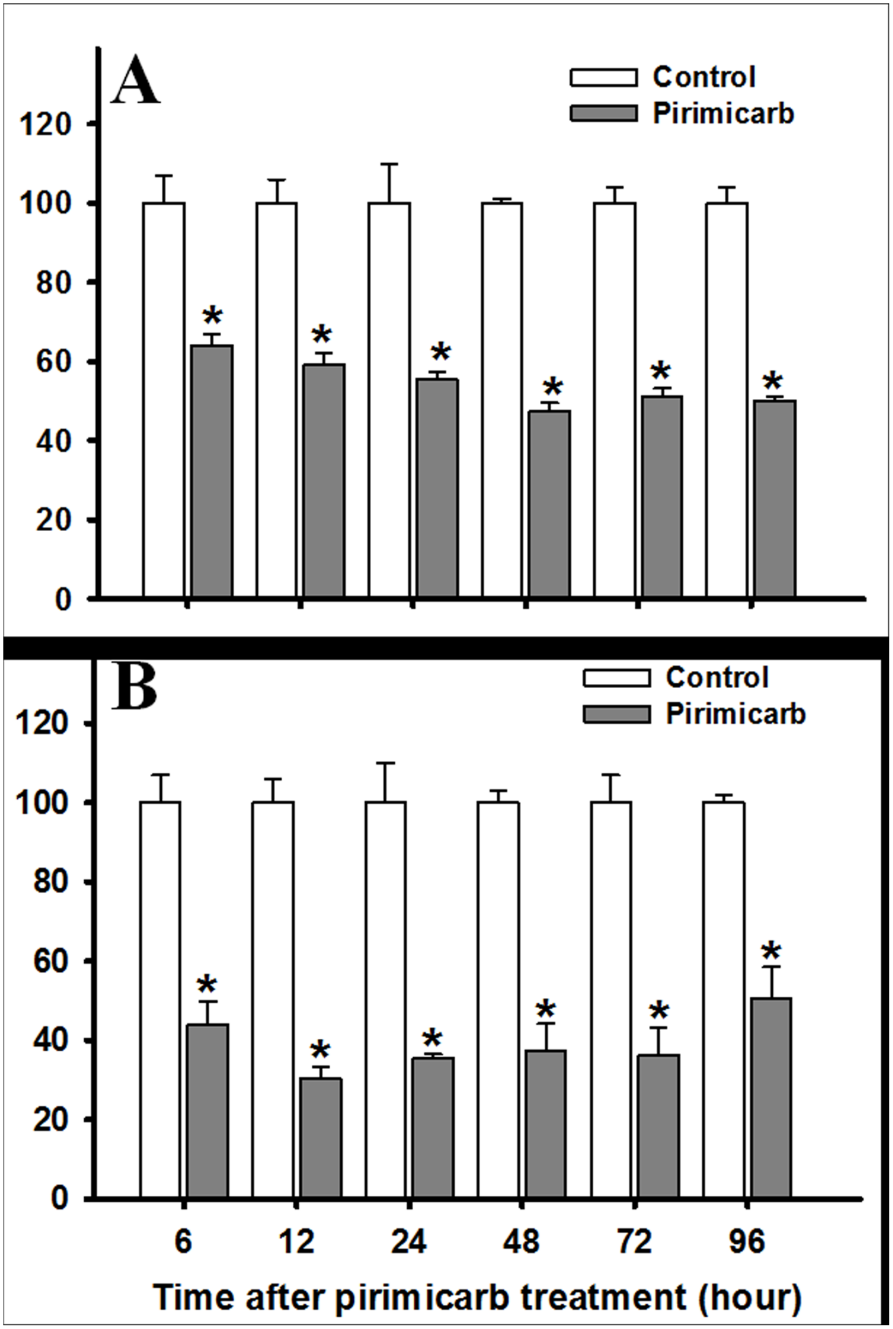
Effects of LC_25_ of pirimicarb on AChE activities of *R*. *padi* (A) and *S*. *avenae* (B) from the parent generation. AChE activities were expressed as nmol of ATCh hydroplyzed/min/mg protein. The results are presented as the mean and standard errors of three replicates. Asterisk on the bars of the histogram indicate significant differences between treatment and respective control (t-test) at *P* < 0.05.

We evaluated the effect of LC_25_ of pirimicarb on the total AChE enzyme activity. Our study showed that exposure by LC_25_ of pirimicarb significantly reduced the AChE activity of *R*. *padi* and *S*. *avenae* when compared to those of their respective control. The AChE activity in pirimicarb-exposed *R*. *padi* decreased up to 52.5% (when compared to those of the control) after 48 h post-treatment (Fig 2A). The AChE activity in pirimicarb-exposed *S*. *avenae* decreased by up to 69.6% (when compared to those of the control) after 12 h post-treatment (Fig 2B).

## Discussion

Sublethal effects of pesticides on arthropods may be negative or positive [11], e.g. positive effects may occur through hormesis [42]. Due to the variable distribution and continuous degradation of active ingredients on plants after initial pesticide applications [36], arthropod populations may be exposed to sublethal concentrations of pesticides. Therefore, research on sublethal effects of pesticides on target pests is of great importance for increasing their rational use, e.g. help design rational pesticide-based IPM (Integrated Pest Management) methods that would delay the development of insecticide resistances in pests. Such studies may also help shedding light on potential for insecticide-mediated shift in ecological dominance between species [43, 31]. In China, the carbamate pesticide pirimicarb is widely used in wheat fields against the aphid pest *R*. *padi* and *S*. *avenae*. Therefore, it may also cause a range of sublethal effects on *R*. *padi* and *S*. *avenae* besides its initial lethal effect; thorough knowledge of these possible effects would help optimize the IPM in wheat crops in China.

The lethal and sublethal effects of pirimicarb have been investigated on many arthropods, e.g. on *Aphidius ervi* [44], *Myzus persicae* [45] and *Coccinella undecimpunctata* [46]. Sublethal effects such as reductions in reproductive capacity and longevity could result in negative impacts on insect population growth [11]. In our study, we found no significant difference in longevity of *R*. *padi* parent generation between the treated and control group. However, the fecundity of parent generation of *R*. *padi* exposed to pirimicarb (LC_25_) was significantly lower than those in the control (Table 2). These results were consistent with previous studies on other carbamate insecticides, such as *Plutella xylostella* exposed to sublethal concentrations of carbaryl [47], *Choristoneura fumiferana* treated with sublethal concentration of aminocarb [48] and *Hippodamia undecimnotata* treated with sublethal concentration of carbofuran [49]. Such effect on adults may result from disturbances in the neurosecretory system caused by the LC_25_ of pirimicarb. The reproduction process is largely regulated by neurohormones in arthropods and neurohormonal imbalance resulting from insecticide poisoning may affect normal reproductive functions [50]. By contrast, the longevity and fecundity of parent generation of *S*. *avenae* were not affected by exposure to the LC_25_ of pirimicarb (Table 2). Similar lack of sublethal effect of pirimicarb has been reported by Cabral et al. (2008) [46] studying *C*. *undecimpunctata*, and by He et al. (2013) [51] assessing sublethal effects of carbosulfan on the fecundity of *Bemisia tabaci*. These contrasting effects stress the need to assess sublethal effects of pesticides on the specific biological models considered for further IPM optimization. In the present study, we observed contrasting results despite studying sublethal effects in two species that were (i) closely related in phylogeny [52] and (ii) that shared the same host plant (wheat). The contrasting effects of pirimicarb may not prompt an insecticide-mediated shift in ecological dominance between the two species: main demographic parameters of *R*. *padi* remained higher than these of *S*. *avenae* despite the negative effects of LC_25_ of pirimicarb on the former (though further studies on this issue are needed because effects of pirimicarb might add up to other biotic and/or abiotic effects in field conditions).

Transgenerational effects were observed in the F1 generation of *R*. *padi* when parents were exposed to LC_25_ of pirimicarb. We found that the overall juvenile development of *R*. *padi* was longer than those in the control. This result was consistent with an earlier study demonstrating that larvae development of *C*. *fumiferana* was significantly extended after exposure to sublethal concentration of carbaryl [48]. Moreover, traits like the duration of generation, reproductive period and adult longevity were also longer than those of the control (Fig 1A). Similar effect, i.e. increased adults longevity in offspring from parents previously exposed to a pesticide, was also observed in *P*. *xylostella* exposed to sublethal concentrations of carbaryl [47] and *Anopheles stephensi* exposed to sublethal concentrations of propoxur [53]. Moreover, the longer reproductive period in treated group led to an average fecundity (per reproductive day) significantly lower than in the control group; there was no significant difference was observed on total fecundity (Table 3). As previously observed in case of individuals in the parent generation, the sublethal effects of the pirimicarb on *R*. *padi* were not found in *S*. *avenae* (Fig 1B).

The demographic parameters in pirimicarb-exposed and control aphid populations provided insight on possible effects of pirimicab on *R*. *padi* and *S*. *avenae* at the generational scale. After exposure of parents to pirimicarb (LC_25_), the *r*_*m*_ values of *R*. *padi* from the F1 generation was lower than those of the control group, whereas both the *T* and *Dt* values were higher than those of the control. In addition, no significant effects on these parameters were observed when *S*. *avenae* exposed to LC_25_ of pirimicarb. This suggests that the LC_25_ of pirimicarb may slow down population growth of *R*. *padi* but *S*. *avenae*.

Hormesis refers to possible enhanced performances of individuals occurring at low levels of exposure to toxic agents that are harmful at high levels of exposure [42]. Pesticide-induced hormesis may be an alternate mechanism explaining pest resurgences; this is a serious problem in agriculture [54]. Hormesis in insects exposed to sublethal concentrations of insecticides has been documented for several taxa and compounds [55]. In the present study, we assessed whether hormesis effects, especially on fecundity and aphid population growth-related traits, may occur in *R*. *padi* and *S*. *avenae* during and/or after exposure (for parent and F1 generation, respectively) to a low dose of pirimicarb. The parent generation of *R*. *padi* exposed to LC_25_ pirimicarb had significantly lower numbers of nymphs produced, and most of aphid development-related traits recorded in F1 generation showed a slow-down development (e.g. increased duration of pre-reproductive period). LC_25_ pirimicarb had no significant effect on the fecundity and longevity in parent generation and various biological traits in F1 generation of *S*. *avenae*. Therefore, no stimulatory effect on fecundity was induced by LC_25_ pirimicarb in *R*. *padi* and *S*. *avenae*, and no resurgence may occur in *R*. *padi* and *S*. *avenae* when exposed to low concentrations of pirimicarb.

AChE is an important biochemical marker in ecotoxicology. In our study, AChE activities in survival aphids provided a measure of sublethal effects of pirimicarb. The results demonstrated that the AChE activity of *R*. *padi* was reduced after exposure to pirimicarb, and the similar trend of AChE activity was observed in *S*. *avenae*. These results were in concordance with the previous studies on organophosphate insecticdes which have same mode action with carbamate insecticides, e.g. for *Micromus tasmaniae* when exposed to azinphos-methyl and methyl parathion [24] and when exposed to diazinon and chlorpyrifos [56]. AChE activity of *Helicoverpa armigera* decreased after exposure to LD_10_ of phoxim [57] and exposure to dimethoate induced an inhibition of AChE activity in both *M*. *tasmaniae* and *R*. *padi* [25]. Moreover, the specific activity of AChE in *M*. *persicae* was reduced significantly when treated with different sublethal doses of imidacloprid [58].

In summary, the present study indicated that a low concentration of pirimicarb would affect the fecundity of *R*. *padi* of parent generation, and would also trigger biological traits variation at the F1 generation of *R*. *padi*. In addition, no stimulatory effects on fecundity were observed in parent and F1 generation of *S*. *avenae* and *R*. *padi*. In practice, these findings under laboratory conditions highlighted the importance of sublethal effects on aphids and how they may translate to population dynamic in the field. Therefore, our study provided evidence for promoting the continued use of pirimicarb for wheat aphid control.
